# Cerebral Fat Embolism Syndrome

**DOI:** 10.31662/jmaj.2024-0345

**Published:** 2025-01-31

**Authors:** Daichi Motomura, Tomotaka Takanosu

**Affiliations:** 1 Department of Emergency and Critical Care Medicine, Jichi Medical University, Shimotsuke-shi, Japan

**Keywords:** fat embolism, consciousness, fracture

A 72-year-old man was presented to our hospital after falling. He was alert upon arrival, and diagnostic tests confirmed a fracture on the left femoral shaft (Figure 1). He was admitted to our hospital. On the 2^nd^ day of admission, he suddenly developed altered consciousness and hypotension. He showed no signs of respiratory failure or skin manifestation. No intracranial abnormalities were identified in computed tomography performed on the same day. On the 3^rd^ day of admission, the patient’s condition worsened, progressing to a comatose state. Brain magnetic resonance imaging revealed a star field pattern and diffuse microhemorrhages (Figure 2, 3, 4 and 5). Therefore, the patient was diagnosed with fat embolism syndrome. Owing to hemodynamic instability, internal fixation of the femoral shaft fracture was performed on the 6^th^ day of admission. His level of consciousness gradually improved, and he was transferred to a rehabilitation facility.

**Figure 1.**
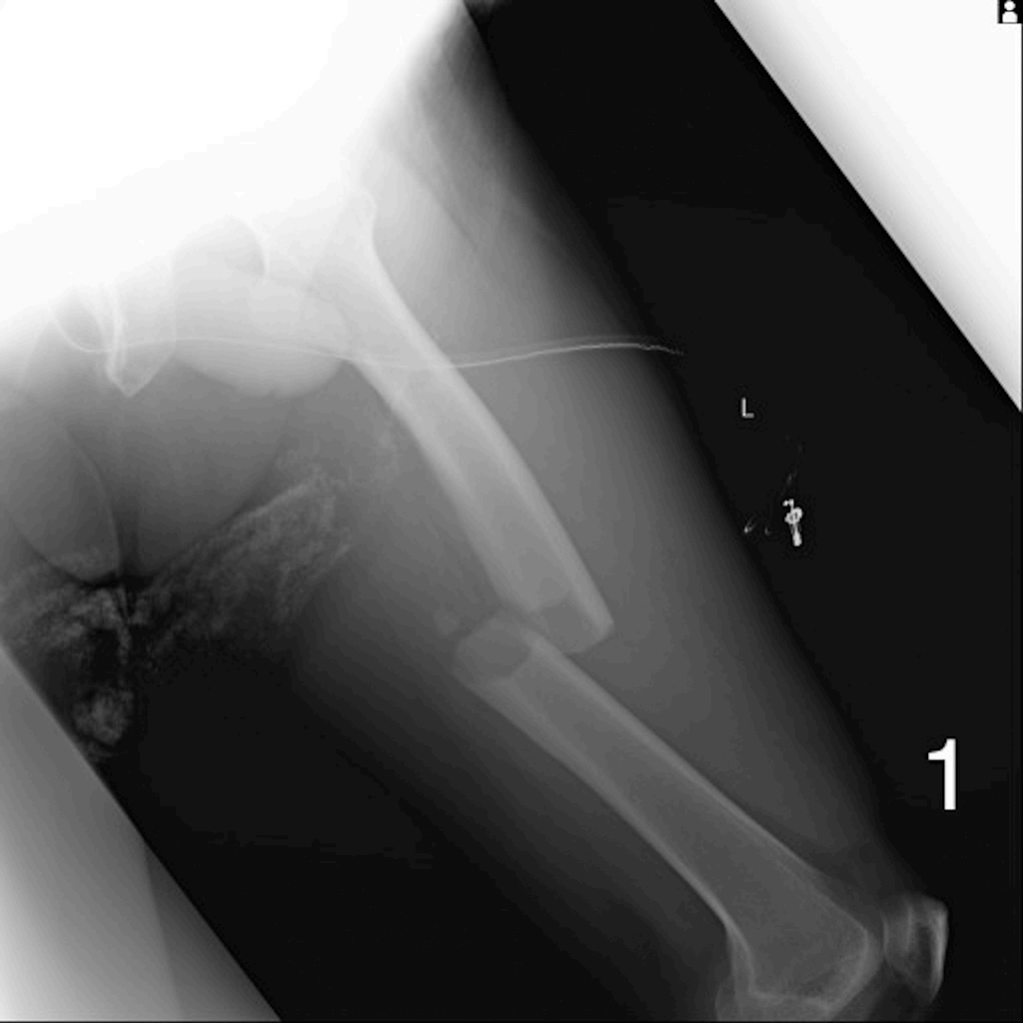
Left femoral shaft on 1^st^ day.

**Figure 2.**
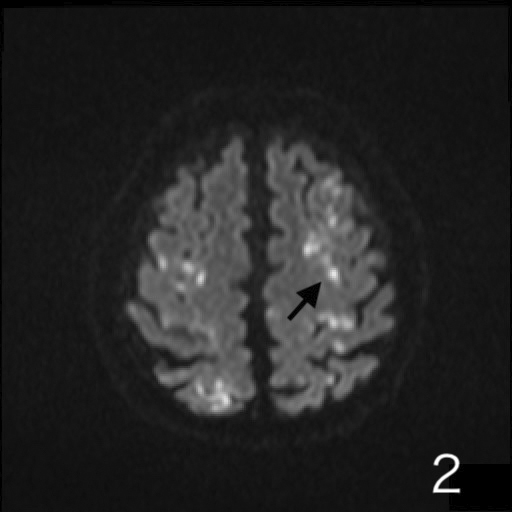
Axial DWI images on 3^rd^ day showing scattered hyper intense signal consistent with starfield pattern at cerebral hemisphere.

**Figure 3.**
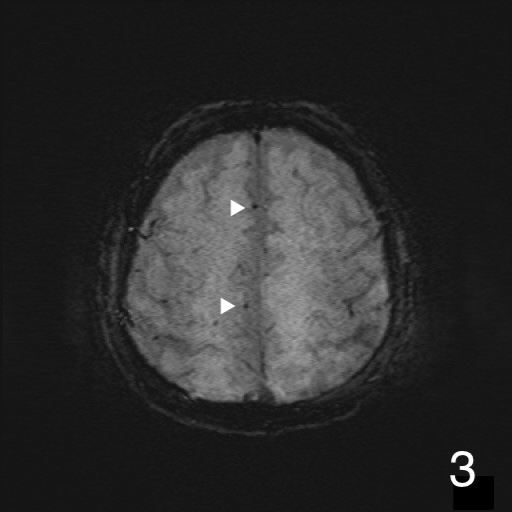
Axial SWI images on 3^rd^ day showing numerous petechial hemorrhages at cerebral hemisphere.

**Figure 4.**
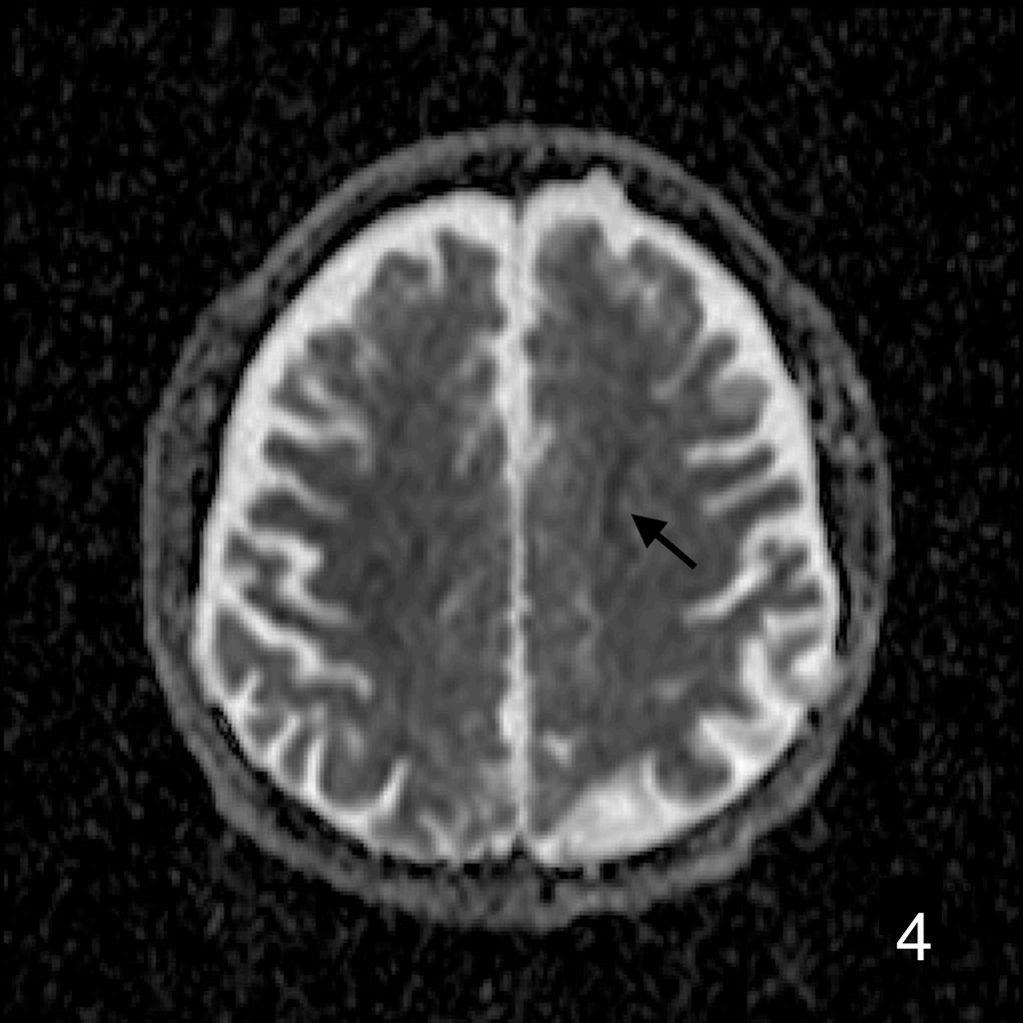
Axial DWI-ADC images on 3^rd^ day shows restriction at cerebral hemisphere.

**Figure 5.**
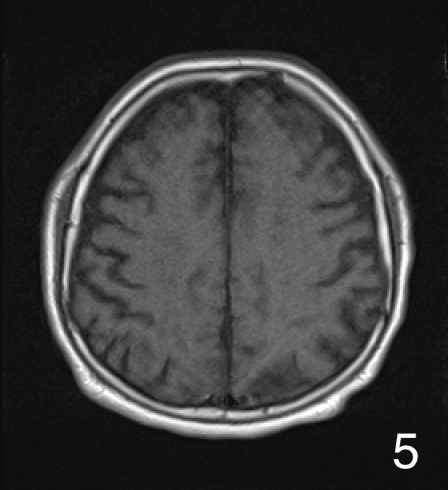
Axial T1-tra images on 3^rd^ day.

## Article Information

### Conflicts of Interest

None

### Author Contributions

Daichi Motomura wrote the first draft of the manuscript, and all authors commented on the previous versions of the manuscript. All the authors have read and approved the final version of the manuscript.

### Informed Consent

Informed consent was obtained by the opt-out method prescribed by the hospital.

